# Opportunistic Sensor Data Collection with Bluetooth Low Energy

**DOI:** 10.3390/s17010159

**Published:** 2017-01-23

**Authors:** Sergio Aguilar, Rafael Vidal, Carles Gomez

**Affiliations:** Universitat Politècnica de Catalunya/Fundació i2Cat, C/Esteve Terradas, 7, 08860 Castelldefels, Spain; saguilardevel@gmail.com (S.A.); rafael.vidal@entel.upc.edu (R.V.)

**Keywords:** Bluetooth Low Energy, Bluetooth Smart, opportunistic data collection, sensor networks, modeling, performance evaluation, beacons, Internet of Things

## Abstract

Bluetooth Low Energy (BLE) has gained very high momentum, as witnessed by its widespread presence in smartphones, wearables and other consumer electronics devices. This fact can be leveraged to carry out opportunistic sensor data collection (OSDC) in scenarios where a sensor node cannot communicate with infrastructure nodes. In such cases, a mobile entity (e.g., a pedestrian or a vehicle) equipped with a BLE-enabled device can collect the data obtained by the sensor node when both are within direct communication range. In this paper, we characterize, both analytically and experimentally, the performance and trade-offs of BLE as a technology for OSDC, for the two main identified approaches, and considering the impact of its most crucial configuration parameters. Results show that a BLE sensor node running on a coin cell battery can achieve a lifetime beyond one year while transferring around 10 Mbit/day, in realistic OSDC scenarios.

## 1. Introduction

Sensor nodes are being deployed worldwide to enable smart environments, whereby resources can be efficiently managed and/or human life quality can be enhanced. In some scenarios, such as homes or industrial automation, network infrastructure is typically available for the sensor nodes, therefore whenever the latter need to send their obtained data, some device must be ready to receive (and maybe forward) the data [1,2]. However, there exist other scenarios where network infrastructure is expensive, or it is hard to deploy for practical reasons, such as smart cities, agricultural ecosystems, developing areas, etc. [3,4,5]. In those, as long as latency requirements for the collected data are loose, opportunistic sensor data collection (OSDC) carried out by a mobile entity may be more suitable. On the other hand, OSDC can also be viewed as a backup or disaster recovery option for data collection when infrastructure fails, similarly to the role of opportunistic networking to provide communications means after disaster recovery [6].

Useful mobile entities for OSDC include pedestrians, and vehicles such as public buses or even drones. Such mobile entities may approach an isolated sensor node on purpose (e.g., an agriculturist intentionally walking, or flying a drone, close to the sensor node location) or due to the path they naturally follow (e.g., the daily route to work for a pedestrian or the scheduled route of a public bus). 

For the sake of flexibility and seamless, low-cost operation, communication between the sensor node and the mobile entity requires the use of a wireless technology commonly or easily available on the mobile entity equipment. At the same time, such technology should allow low-power operation for the sensor node, which typically is not mains-powered and has a limited energy source (e.g., a battery). A major candidate technology that fulfills such requirements is Bluetooth Low Energy (BLE) [7,8]. In fact, since BLE is the wireless low power technology best established in the mobile market (massively implemented e.g., in smartphones, tablets, and wearables), it is a natural choice for developing an OSDC solution. Competitors such as Ant/Ant^+^ are not supported by any standardization body, while other standardized technologies are today far from competing with BLE in this specific domain. IEEE 802.15.4 could be an option with the release of modular smartphones [9], but their adoption seems far in the near future. Secondly, the work conveyed in order to extend Wi-Fi to meet the requirements of IoT [10,11] is focused on infrastructure-based scenarios. Finally, Visible Light Communication (VLC) technologies are progressing in their standardization [12], but their intrinsic characteristics make them only well suited basically for indoor and short range communications.

Furthermore, BLE is emerging as a key enabler of the Internet of the Things (IoT). The methods to run IPv6 over BLE have been standardized [13], allowing the use of BLE devices such as smartphones as gateways [14]. On the other hand, there exist proposals to extend BLE for mesh network topologies which are progressing towards standardization [15,16]. 

With regard to market products, the BLE ecosystem is growing [17]. The recent family of products called beacons, designed for opportunistic data collection, has achieved remarkable momentum. Beacons can advertise identifiers (e.g., URLs) and sensed data [18].

Considering all the above, BLE is a promising technology for OSDC. It is thus crucial to know the achievable performance of BLE in such scenario for the design, planning and practical use of OSDC solutions. However, use of BLE for OSDC has only been considered to a limited extent [19,20,21]. To our best knowledge, a detailed, comprehensive characterization of BLE for OSDC is not available in the literature.

In this paper we provide an analytical and experimental study of the performance and trade-offs of BLE as a technology for OSDC. We consider the two main BLE modes of operation, and the impact of its most crucial configuration parameters. Results show the feasibility of using BLE for OSDC: a sensor node running on a coin cell battery can achieve a lifetime beyond one year while transferring around 10 Mbit/day, in realistic OSDC scenarios. 

The remainder of the paper is organized as follows: Section 2 reviews related work. Section 3 provides an overview of BLE, highlighting its main mechanisms and configuration parameters, and describes the two identified approaches that can be used to perform OSDC. Section 4 analytically models the achievable performance of the previously described approaches for OSDC with BLE. Section 5 provides evaluation results using both the analytical models, as well as empirical tools, and Section 6 concludes the paper.

## 2. Related Work

Despite the current market and standardization current status described in Section 1, which is favourable for using BLE for OSDC, to the best of our knowledge, there are very few published papers related with this topic. These are reviewed in the next subsection, highlighting their relationship with our work. Then, in a second subsection we survey the extensive literature on BLE performance, focusing on BLE capabilities that are interesting for OSDC. 

### 2.1. Opportunistic Sensor Data Collection Using Bluetooth Low Energy

The authors of [19] defined the OSDC problem in a general fashion and mentioned BLE as a candidate technology, but not evaluated, in a scenario where the mobile entities are smartphones. The feasibility of using BLE for OSDC has been demonstrated through a proof of concept, also with smartphones as mobile entities [20]. The work included a limited study with fixed BLE parameters and mode configuration. Results showed the low impact of BLE activity on the power consumption of the mobile entity (that was able collect data for 3.5 days, with a maximum throughput of 83.33 kbit/s, a round-trip time between 30 ms and 50 ms, and a coverage range up to 16 m). As will be explained in the following subsection, BLE performance can be much better.

Finally, Unmanned Aerial Vehicles (UAVs) have been proposed to collect data from a WSN using BLE [21]. The work includes an analytical model of the power consumption of sensor nodes, based on published information of commercial Systems on Chip (SoCs). To our best knowledge, this is the only published study that evaluates the performance for a specific OSDC variable: the contact time. However, the model only considers the maximum and minimum values of discovery and connection times for one of the two BLE modes of operation, leaves out important performance parameters like the amount of collected information per contact interval, and does not evaluate the impact of the main BLE parameters on performance. 

### 2.2. Bluetooth Low Energy Performance

The trade-off between energy consumption, latency, network size, and throughput for one of the BLE modes (namely, connection-based mode) is studied in [8], providing experimental, theoretical and simulation results. These results show that using a coin cell battery, lifetimes of up to 14.1 years can be achieved. The average latency of one round-trip for very low BER values (e.g., 10^−6^) is smaller than 2 ms, being below 2 s in the worst conditions considered (BER = 10^−3^). The number of sensor nodes (slaves in BLE terminology) simultaneously connected to a mobile entity (a master in BLE terminology) could be up to 5917. The maximum experimental throughput at the application layer is 58.48 kbps. These performance results provide a solid foundation to propose an OSDC connection-based BLE solution.

The BLE device discovery mechanism has been deeply studied [22,23,24]. An analytical model validated by simulation is presented [25]. Results are useful to understand the behaviour of an OSDC scenario with multiple devices performing discovery at the same time. However, the work does not include any empirical study, node lifetime and amount of information exchanged are out of scope, and the connection phase is not considered.

With regard to sensor node energy consumption, authors in [23] develop a BLE energy consumption analytical model validated with empirical measurements. Since the study does not focus on the OSDC use case, the impact of some relevant aspects on performance, such as the contact time or the maximum amount of information that can be exchanged, are not considered.

BLE throughput has been evaluated in several works. The maximum application layer throughput reported in experimental studies is between 60 and 80 kbit/s [8,20,26]. These low values are mainly due to limited hardware and setup capabilities. Throughput at the link layer is studied analytically and empirically in [27]. The maximum throughput obtained empirically is 122.6 kbit/s, achieved with the connection-based approach. In this way, two interesting contributions of our work are the achievement of a greater empirical maximum of 156.5 kbit/s, and the fact that this value remains almost constant until distances of approximately 200 m. As explained in Section 5.5.1, we obtained this maximum with an aggressive setup of the BLE modules employed [28], and it far exceeds the one announced by their manufacturer (i.e., 60 kbit/s), but matches perfectly the analytically obtained one. To the best of our knowledge it is the highest empirical BLE throughput published in the literature.

Because OSDC suffers from the non-static nature of its nodes, it is interesting to know the behaviour of BLE in dynamic scenarios. This is empirically shown in [29], using off-the-shelf smartphones in an Inter-Vehicular Communications (IVC) scenario. Maximum communication ranges beyond 100 m and robust connection up to 50 m are obtained. The authors show the connection time between two vehicles as a function of their relative speed. In an urban scenario, contact times between 50 s and 10 s, and packet transmissions between 464 and 85 packets, are achieved with speeds from 20 km/h to 60 km/h, respectively. Finally, an interference test is conducted where BLE communications coexist with Wi-Fi ones, concluding that BLE is interference-resilient. These results empirically demonstrate the capability of BLE for data collection even in challenging scenarios. However, they cannot be extended because a very specific testbed setup is employed. In this way, this paper completes and expands that work.

## 3. Using Bluetooth Low Energy for Opportunistic Sensor Data Collection

In this section, we first provide an overview of BLE [7], highlighting its mechanisms most relevant for this work. Then we identify and describe the two main approaches that may be used to opportunistically collect data from a BLE-enabled sensor node.

### 3.1. Bluetooth Low Energy Overview

BLE defines a complete protocol architecture intended to enable low-power communication [7,8,14,30]. This subsection describes the Physical Layer and the Link Layer, which are the main layers in the context of this work. 

#### 3.1.1. Physical Layer

At the Physical Layer, BLE defines 40 Radio Frequency (RF) channels in the Industrial Scientific Medical (ISM) 2.4 GHz band. Such channels are divided into three advertising channels, which are used for broadcasting purposes, and 37 data channels, which allow bidirectional message exchange between two connected devices. The physical data rate is 1 Mbit/s.

#### 3.1.2. Link Layer

In BLE, communication between two devices may take place following two main Link Layer interaction patterns: the two devices may act as: (i) advertiser and scanner, whereby the advertiser unidirectionally transmits data that can be received by the scanner, or as (ii) master and slave, whereby the two devices have established a connection and may exchange data bidirectionally. 

An advertiser transmits advertising packets through advertising channels within time periods called advertising events. The time between two consecutive advertising events is equal to *advInterval* + *advDelay*, whereby *advInterval* has a fixed value that may be configured between 20 ms and 10.24 s (for non-connection-oriented advertising packets, *advInterval* ranges between 100 ms and 10.24 s), and *advDelay* is a random variable uniformly distributed between 0 and 10 ms, intended to avoid possible harmful synchronization effects with other advertisers. In one advertising event, an advertiser transmits an advertising packet through one, two or the three advertising channels. 

In order to enable bidirectional data exchange between two devices, they must establish a Link Layer connection. To this end, one of the devices has to advertise that it is connectable. The other device, called initiator, listens for such advertisements. When the initiator detects the presence of a connectable advertiser, it may send a Connection Request message to that advertiser, which has to be listening for such possible incoming messages. When a Connection Request message is received by the advertiser, a Link Layer connection has been established, and both devices may communicate using data channels. Within a connection, the former advertiser and initiator will play the slave and master roles, respectively. The Connection Request message includes the parameters that govern a connection. After the transmission of the Connect Request message, a wait time of 1.25 ms elapses, and the master may delay the transmission of its first data packet up to *TransmitWindowSize* time.

Within a connection, a slave is assumed to be by default in sleep mode to save energy, and turn on its radio interface periodically for incoming packets from the master. Communication between a master and a slave takes place in time intervals called connection events. At the beginning of a connection event, the master sends a packet to the slave, which must respond to the master. After that, the exchange of messages may continue if any of the devices has more data to transmit. From the end of the transmission of a packet until the start of the next one, an Inter Frame Space (IFS) of at least 150 μs must be guaranteed. The packet flow is controlled by means of a stop-and-wait mechanism based on cumulative acknowledgments, with error recovery assisted by negative acknowledgments. When the two connected devices have sent all their pending data, the connection event will be closed. Connection event closure occurs also if certain errors affect the communication (the reception of two consecutive packets with errors, or an error affecting the address field of a packet) or if the next connection event start is imminent (i.e., more data cannot be sent and acknowledged within the remaining time before the next connection event). 

The time between the start of two consecutive connection events is given by a parameter called *connInterval*, which may adopt values from 7.5 ms to 4 s. Another relevant parameter is *connSlaveLatency*, which states a number of consecutive connection events (between 0 and 499) during which the slave may skip listening to the master to save energy. The connection health is tracked by running a timer that accounts for the time since the last packet was received. If that time exceeds the *connSupervisionTimeout* parameter (between 100 ms to 32 s, and equal to or greater than *connInterval*), the connection is assumed to have failed. 

### 3.2. Approaches for Opportunistic Sensor Data Collection with Bluetooth Low Energy

**Figure 1:**
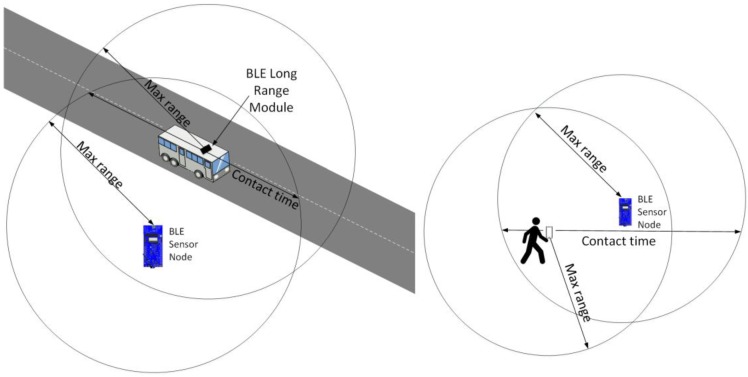
Illustration of OSDC concept examples, where the mobile entity is a bus (**Left**) or a pedestrian (**Right**). The mobile entity is equipped with a BLE device and collects data from the sensor node during the contact time.

In this paper we consider OSDC, where a mobile entity falls in the coverage range of the BLE-enabled sensor node (and vice versa) during a certain contact time. We assume that the sensor node has accumulated data from sensor readings taken over a certain period, and when a contact takes place, the mobile entity collects (a subset of) the accumulated data from the sensor node. Figure 1 illustrates two examples of the described OSDC paradigm, where a bus or a pedestrian carrying a BLE device (e.g., a smartphone) play the mobile entity role.

**Figure 2:**
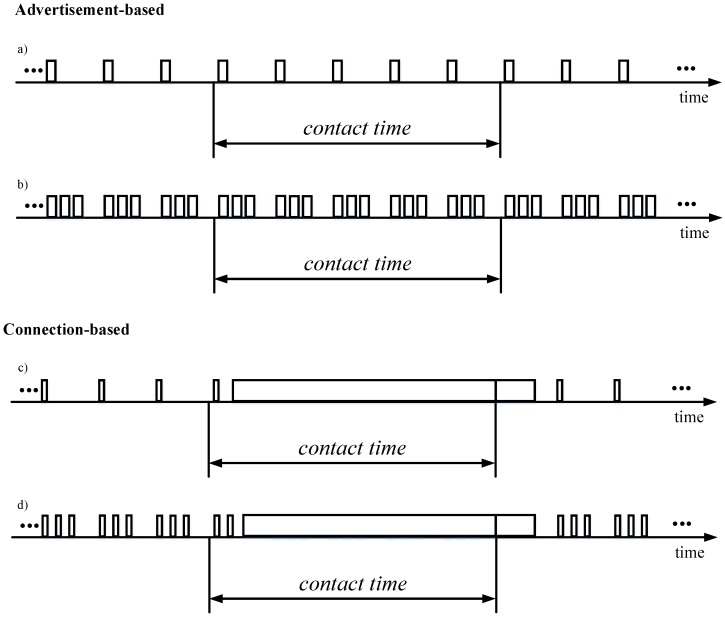
Illustration of the two main OSDC approaches with BLE, for the two different advertisement settings in each one. (**a**) advertisement-based approach with one advertising packet per advertising event; (**b**) advertisement-based approach with three advertising packets per advertising event; (**c**) connection-based approach with one advertising packet per advertising event; (**d**) connection-based approach with three advertising packets per advertising event.

Considering the functionality offered by BLE, we identify two main approaches for OSDC with BLE: advertisement-based and connection-based. The first one relies on the use of the sensor node as an advertiser. In this case, the advertisements sent by the sensor node are used as a channel for transporting information. If the size of the data object to be transmitted exceeds the payload of an advertising packet, we assume that the object is fragmented in units that fit the maximum advertising packet payload size. The sensor node sequentially transmits the different fragments, and after the last one, it starts transmitting again the same sequence of fragments. The specific details of how such a mechanism would work are out of the scope of this paper, since our aim is to determine the capacity and limits of OSDC with BLE. If the data object to be transmitted by the sensor node fits a single advertising packet, the sensor node continuously transmits that object in its advertising packets. The mobile entity listens for the advertising packets sent by the sensor node. The former will be able to receive the advertising packets sent during the contact time. The sensor node may send from one to three advertising packets in each advertising event (Figure 2a,b, respectively). In the second case, the same advertising packet is sent via the three advertising channels in each advertising event. While this option provides greater frequency diversity, it leads to greater energy consumption by the sensor node. Further details on the procedures and modeling of the advertisement-based approach can be found in Section 4.1.

The second approach for opportunistic sensor node data collection is based on the establishment of a connection between the sensor node and the mobile entity, as soon as the contact between both takes place (Figure 2c,d). In this approach, the sensor node by default transmits advertising packets to announce that it is a connectable device. Advertising packets in this approach do not carry user data, and thus have a shorter size than the ones used in the advertisement-based approach, as illustrated in Figure 2. As in the advertisement-based approach, between one and three advertising packets can be sent in each advertising event (Figure 2c,d, respectively), and the sensor node consumes a greater amount of energy with the last option. When the mobile entity receives one of the advertising packets, it initiates connection establishment by sending a Connection Request to the sensor node. Once the connection is established, the sensor node transmits the accumulated data to the mobile entity. Once the two endpoints fall out of each other’s range, and after the supervision timer of the sensor node triggers connection failure detection, the sensor node returns to sending advertising packets to announce its connectability. Further details on the procedures and modeling of the connection-based approach can be found in Section 4.2.

## 4. Modeling the Performance of Bluetooth Low Energy for Opportunistic Sensor Data Collection 

This section provides analytical models of crucial performance parameters for OSDC with BLE: sensor node current consumption and lifetime, and the maximum amount of collected data per contact interval. We assume a non-ideal channel, with uncorrelated bit errors. The section is divided in two subsections, which offer the aforementioned models for the advertisement-based and connection-based approaches, respectively.

### 4.1. Advertisement-Based Approach 

#### 4.1.1. Sensor Node Current Consumption and Lifetime

**Figure 3:**
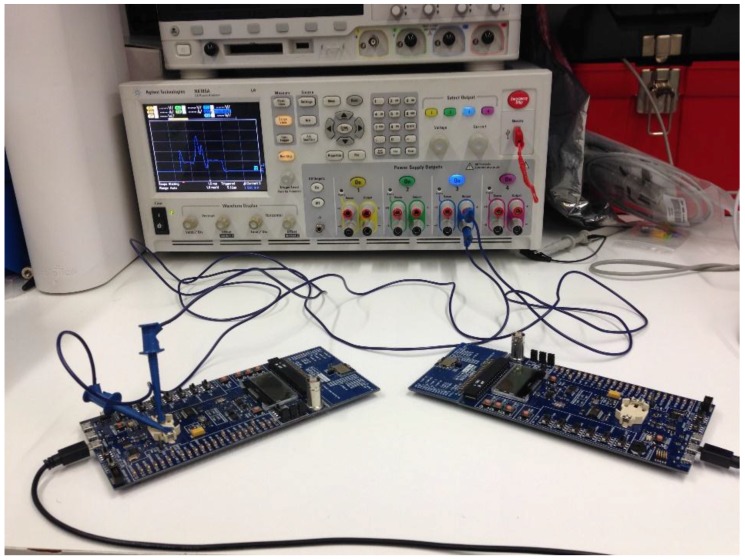
Experimental setup for current measurements of the BLE121LR modules using an Agilent N333 power analyzer. The module at the left works as a slave that connects to the module at the right, which operates as a master.

Our first goal is modeling the average current consumption of a sensor node in the advertisement-based approach, denoted *I_avg_adv_*. Computing the average current consumption of a device requires knowledge of the different states it traverses, and the duration and the current consumed in each state. With the aim to capture a realistic behavior in our model, and without loss of generality, we derive the model from measurements on a real BLE platform. The measurement setup is shown in Figure 3. Since we aim at exploring the capability limits of OSDC for BLE, we select the BLE121LR platform from Bluegiga [28] as our reference platform for the model. This platform implements BLE as per the Bluetooth 4.0 specification, and provides a range of around half kilometer, which is longer than that of typical BLE modules. Note that performance of the selected platform in terms of current consumption is very similar to that of other BLE platforms [31,32,33,34], although BLE121LR exhibits a greater current consumption for transmit and receive states to achieve significantly longer range than typical BLE platforms. In this regard, using BLE121LR for the study in this paper allows us to provide an upper bound in terms of maximum amount of collected data per contact interval, and a lower bound on the sensor node lifetime that can theoretically be achieved.

Because the behavior of the BLE sensor node in the advertisement-based approach is periodic, we model its current consumption during one such period. Each period comprises one advertising event, and otherwise the device is in sleep mode. Time and current consumption measurement results provided in this section are obtained from 10 measurements for a period. We found negligible differences within each set of 10 measurements, and results are well aligned with the ones also provided by the manufacturer [28]. 

**Figure 4:**
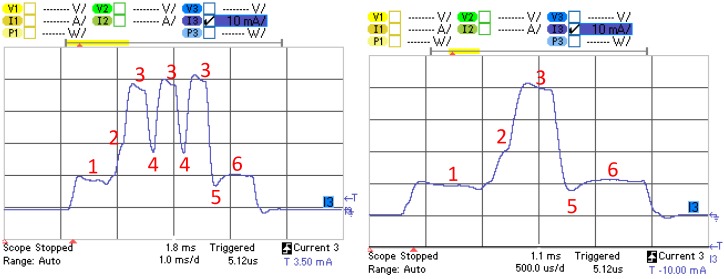
Current consumption profile of an advertising event for the BLE121LR platform operating as a non-connectable advertiser. Three-advertisement (leftmost) and single-advertisement (rightmost) advertising events are shown.

Figure 4 illustrates the current consumption profile of an advertising event corresponding to a BLE121LR platform for the advertisement-based approach, that is, as a non-connectable advertiser. The different states, their description, and the variables used to define their duration and current consumption, are shown in Table 1. The sensor node is initially in sleep mode, typically consuming a current in the microampere order. When the advertising event starts, the device wakes up (State 1), the radio interface is prepared for activity (State 2), and then the device transmits an advertising packet with a total size of 47 bytes (State 3). If the device sends more than one advertising packet in the advertising event, then it changes the physical channel frequency (State 4), and performs the remaining advertising packet transmissions following the same approach as for the first advertising packet, with the exception that after the last transmission, the radio of the device is turned off (State 5) and finally a postprocessing interval (State 6) takes place before the device returns to sleep mode, in which the device will remain until the start of the subsequent advertising event. Note that in the advertising-based approach, the radio of the sensor node is never in receive mode.

**Figure 5:**
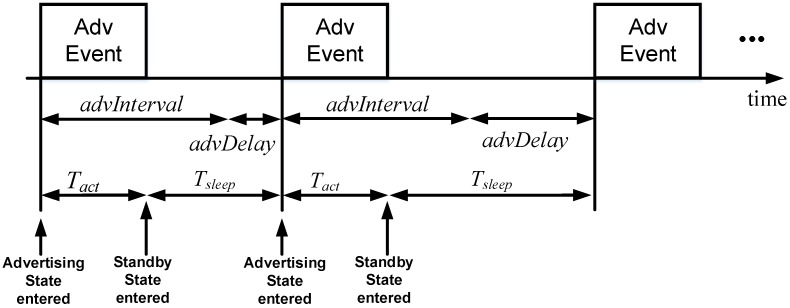
Illustration of variables involved in the calculation of the average current consumption in the advertisement-based approach (*I_avg_adv_*). ‘Adv Event’ refers to an advertising event.

Let *T_act_* and *I_act_* be the duration and current consumption, respectively, of the active part (i.e., where the device is not in sleep mode) of the advertising interval (see Figure 5). Since the average duration of a period is equal to *advInterval* + *E*{advDelay}, *I_avg_adv_* can be calculated as shown in Equation (1):

(1) $$I_{avg\_adv}=\;\frac{T_{act}\cdot I_{act}+T_{sleep}\cdot I_{sleep}}{advInterval+E\left\{advDelay\right\}}$$

where, assuming that *N* denotes the number of advertising packets sent within an advertising interval (with *N* ∈ {1, 2, 3}), *T_act_* can be computed as:

(2) $$T_{act}=T_{wu}+T_{pre}+N\cdot T_{tx}+\left(N-1\right)\cdot T_{ch}+T_{off}+T_{post}$$

and *I_act_* is the average current consumption during *T_act_*:

(3) $$I_{act}=\frac{T_{wu}\cdot I_{wu}+T_{pre}\cdot I_{pre}+N\cdot T_{tx}\cdot I_{tx}+\left(N-1\right)\cdot T_{ch}\cdot I_{ch}+T_{off}\cdot I_{off}+T_{post}\cdot I_{post}}{T_{act}}$$

Note that *T_sleep_* can be obtained as:

(4) $$T_{sleep}=advInterval+E\left\{advDelay\right\}\;-T_{act}$$

Finally, assuming that the sensor node operates on a battery, the sensor node lifetime, denoted *T_lifetime_*, can be derived taking into account the battery capacity, *C_battery_* (expressed in mA·h), as follows:

(5) $$T_{lifetime}=\;\frac{C_{battery}}{I_{avg\_adv}}$$

As a final remark, note that possible communication bit errors do not affect the sensor node average current consumption or the sensor node lifetime in the advertisement-based approach. In fact, transmitting advertisements consumes an amount of energy that is independent of whether those advertisements will suffer communication errors, and it does not require feedback from the receiver.

#### 4.1.2. Maximum Amount of Collected Data per Contact Interval

We next model the expected maximum amount of data, denoted *E*{*L_data_adv_*}, that the mobile entity may collect from the sensor node within a contact interval in the advertisement-based approach.

Let *T_contact_* be the duration of the contact interval. Let *L_adv_non_* and *L_adv_payload_* denote the maximum physical layer size of and the total amount of data that may be transported in a non-connectable advertising packet, respectively. Let *b* denote the Bit Error Rate (BER) of the channel. Then, *E*{*L_data_adv_*}** can be computed as follows:

(6) $$E\left\{L_{data\_adv}\right\}=\frac{T_{contact}}{advInterval+E\left\{advDelay\right\}}\cdot \;L_{adv\_payload}\cdot {\left(1-b\right)}^{L_{adv\_non}}$$

### 4.2. Connection-Based Approach 

#### 4.2.1. Sensor Node Current Consumption

**Figure 6:**
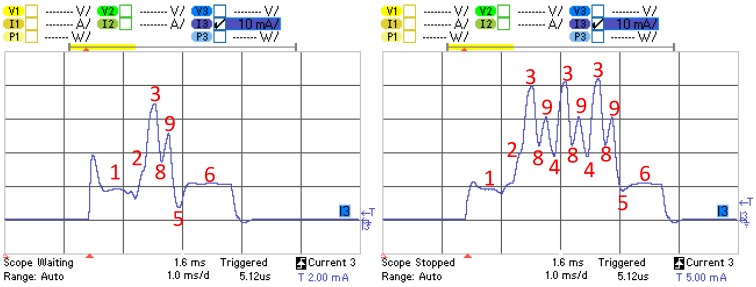
Current consumption profile of an advertising event for the BLE121LR platform, operating as a connectable advertiser. Single-advertisement (**Left**) and three-advertisement (**Right**) advertising events are shown.

We next model the average current consumption of a sensor node in the connection-based approach, on the basis of the current consumption profile of the BLE121LR platform. In this approach, the BLE sensor node operates by default as an advertiser that announces its connectability. Therefore, the sensor node has to stay for a while in receive mode, after the transmission of each advertising packet, for possible incoming Connection Request messages. Figure 6 illustrates the current consumption profile of an advertising event corresponding to a BLE121LR platform for the connection-based approach, that is, as a connectable advertiser. The size of the advertisement packets for the connection-based approach is 23 bytes. The additional states of the connectable advertiser, their description, and the variables used to define their duration and current consumption, are shown in Table 2. The difference with the profile of the non-connectable advertiser shown in Figure 5 lies in States 8 and 9, where after the transmission of an advertisement, the device switches to reception during a short interval (State 8) and stays in reception mode (State 9) before transitioning into the radio off state (State 5) or before the next advertisement transmission (State 3).

When the sensor node and the mobile entity are within range, a connection is established. We assume that the data exchange during the connection takes place until the end of the contact interval, with *connSlaveLatency* = 0, which provides an upper bound on both the current consumption of the sensor node, and the maximum amount of data that may be collected. Once the link between the sensor node and the mobile entity fails, the sensor node returns to advertising upon expiration of the connection supervision timeout. 

**Figure 7:**
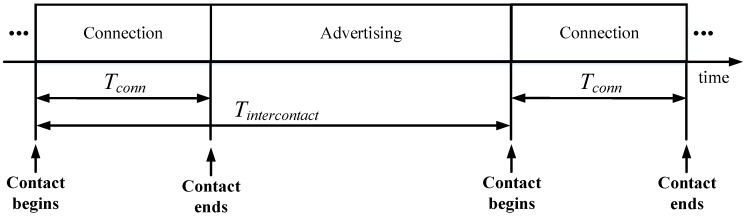
Illustration of time variables involved in the calculation of the average current consumption in the connection-based approach (*I_avg_conn_*).

The average current consumption in the connection-based approach can be computed as follows:

(7) $$I_{avg\_conn}=\;\frac{\left(T_{intercontact}-T_{conn}\right)\cdot I_{adv\_conn}+T_{conn}\cdot I_{conn}}{T_{intercontact}}$$

where *T_conn_* and *I_conn_* denote the duration and average current consumption of the connection-related phase, respectively, *I_adv_conn_* is the average current consumption while the sensor node is advertising, and *T_intercontact_* is the time between two consecutive contact events. Figure 7 shows an overview of the main variables and intervals involved in Equation (7).

Among the variables in Equation (7), *I_adv_conn_* can be computed by using Equation (1), but replacing *T_act_* and *I_act_* by *T_act_connadv_* and *I_act_connadv_*, respectively, which denote the duration and the current consumption of the active part of the advertising period. *T_act_connadv_* can be derived as follows:

(8) $$T_{act\_connadv}=T_{wu}+T_{pre}+N\cdot \left(T_{tx}+T_{tx\_rx}+T_{rx}\right)+\left(N-1\right)\cdot T_{ch}+T_{off}+T_{post}$$

whereas *I_act_connadv_* can be determined as:

(9) $$\begin{array}{c} I_{act\_connadv}\\ =\frac{T_{wu}\cdot I_{wu}+T_{pre}\cdot I_{pre}+N\cdot \gamma +\left(N-1\right)\cdot T_{ch}\cdot I_{ch}+T_{off}\cdot I_{off}+T_{post}\cdot I_{post}}{T_{act\_connadv}} \end{array}$$

where γ is defined as:

(10) $$\gamma =\;T_{tx}\cdot I_{tx}+T_{tx\_rx}\cdot I_{tx\_rx}+T_{rx}\cdot I_{rx}$$

**Figure 8:**
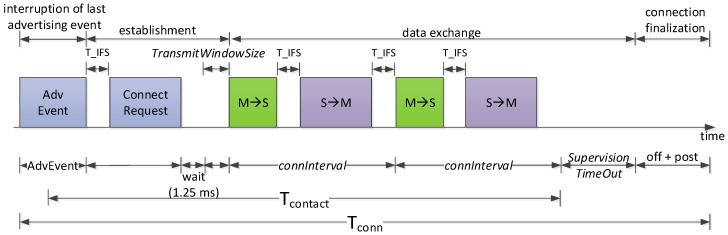
Illustration of the components related with connection establishment, use and finalization. S and M denote Slave and Master, respectively. A round trip exchange comprises a packet sent by the master to the slave, and the response sent by the latter.

On the other hand, *T_conn_* and *I_conn_* comprise the following components: (i) interruption of the last advertising event before the connection; (ii) connection establishment; (iii) data exchange; and (iv) connection finalization after the end of the contact (see Figure 8). We next analyze these four components.

When the mobile entity receives an advertising packet, it then initiates the connection by sending a Connection Request message in response, interrupting the current advertising period. Note that, since bit errors may affect advertising packets or Connection Request messages, *T_conn_* may decrease as a result. However, in this article we consider such decrease to be quantitatively negligible, since we assume *T_contact_* >> *advInterval* (note that otherwise the OSDC system may become impractical), and BER ≤ 10^−3^ (i.e., up to the BER value for which receiver sensitivity is defined as per the BLE standard [30]). 

The connection establishment phase includes the transmission of the Connect Request message (preceded by an IFS interval), the wait time of 1.25 ms, and a time up to *TransmitWindowSize* after which the first data packet will be sent by the master (i.e., the mobile entity). Since we are interested in determining the maximum amount of data that can be collected by the mobile entity, we assume that *TransmitWindowSize* is equal to 0.

Let *T_IFS_*, *T_CReq_*, and *T_wait_* define the IFS duration, the transmission time of the Connect Request message, and the wait time of 1.25 ms, respectively. The time and current consumption of the connection establishment phase are denoted by *T_setup_* and *I_setup_*, respectively, and can be found using the following equations:

(11) $$T_{setup}=T_{IFS}+T_{CReq}+T_{wait}$$

(12) $$I_{setup}=\frac{T_{IFS}\cdot I_{IFS}+T_{creq}\cdot I_{rx}+T_{wait}\cdot I_{wait}}{T_{setup}}$$

Once the connection has been set up, data are exchanged in connection events. Within these, a number of round trip exchanges between master and slave take place (see Figure 8). Let *N_CE_* be the expected number of connection events (of up to *connInterval* duration) that there can be during the contact time. Since there is an initial interval within the contact time, before the first connection event, where connection events cannot take place, *N_CE_* can be computed as follows:

(13) $$N_{CE}=\;\frac{T_{contact}-\left(\frac{advInterval}{2}+\frac{advDelay}{4}+T_{IFS}+T_{CReq}+T_{wait}\right)}{connInterval}$$

(Note: we purposefully do not define *N_CE_* as an integer number, since both the rounded down integer value, as well as the exact value, are needed later, see Section 4.2.2.)

We next calculate the average current consumption within a period of *connInterval* duration, *I_avg_CI_*, which comprises the connection event (composed by a number of round trip exchanges between the master and the slave) plus an inactive part, where the device performs postprocessing operations. First, we compute the average current consumed during a round trip exchange between the master and the slave, *I_RT_*:

(14) $$I_{RT}=\;\frac{I_{MS}\cdot T_{MS}+I_{SM}\cdot T_{SM}+2\cdot I_{IFS}\cdot T_{IFS}}{T_{MS}+T_{SM}+2\cdot T_{IFS}}$$

where *T_MS_* and *T_SM_* are the transmission time of a data channel packet sent by the master to the slave, and by the slave to the master, respectively, and *I_MS_* and *I_SM_* are the current consumption values corresponding to those actions. Note that *T_MS_* and *T_SM_* correspond to *I_rx_* and *I_tx_*. On the other hand, we can determine the round trip duration, *T_RT_*, as:

(15) $$T_{RT}=\;T_{MS}+T_{SM}+2\cdot T_{IFS}$$

Then, in an ideal, error-free scenario, *I_avg_CI_* is given by the following equation:

(16) $$I_{avg\_CI}=\;\frac{\lfloor \frac{connInterval}{T_{RT}}\rfloor \cdot I_{RT}\cdot T_{RT}+\left(\frac{connInterval}{T_{RT}}-\lfloor \frac{connInterval}{T_{RT}}\rfloor \right)\cdot I_{post}\cdot T_{RT}}{connInterval}$$

However, a connection event may be prematurely closed due to certain communication errors (see Section 3.1.2). Within *connInterval*, there will thus be a first active interval where packets are exchanged between the two link endpoints, and a second interval after premature connection event termination, where the sensor node will turn off the radio, go through a postprocessing state, and then will sleep until the start of the next connection interval. In that case, *I_avg_CI_* can be approximated by using the following equation:

(17) $$I_{avg\_CI}=\;\frac{\varphi \cdot I_{RT}\cdot T_{RT}+I_{off}\cdot T_{off}+I_{post}\cdot T_{post}+\left(connInterval-\varphi \cdot T_{RT}-T_{off}-T_{post}\right)\cdot I_{sleep}}{connInterval}$$

where φ denotes the expected number of round trip exchanges before connection event termination within *connInterval*, which can be obtained analytically by using Equation (2) of [30], assuming that bit errors are uncorrelated. Note that the previous equation provides an accurate, but not exact value for *I_avg_CI_* since a further failed round trip exchange may take place in some situations, after the last successful one, leading to a current consumption increase, which will anyway be minor. Another observation is that the above equation is valid for most *connInterval* values, but for very low *connInterval* values (i.e., connInterval < 10 ms), there may not be enough time within *connInterval* for the node to traverse all the states mentioned after connection even termination. In that case, Equation (17) can be rewritten as follows:

(18) $$I_{avg\_CI}=\;\frac{\varphi \cdot I_{RT}\cdot T_{RT}+I_{remainder}\cdot T_{remainder}}{connInterval}$$

where *T_remainder_* and *I_remainder_* denote the duration and average current consumption of the remainder states subsequent to premature connection event termination, before the start of the next connection event. The remainder interval may comprise a partial radio off period, a complete radio off period, or a complete radio off period plus a complete postprocessing period, depending on *connInterval* setting. Note that, for a large majority of *connInterval* values, Equation (17) will be used.

Once the link between the mobile entity and the sensor node has got broken, a time of *connSupervisionTimeout* passes until the latter detects the connection failure and returns to advertising. We assume that while the supervision timer is running, since the sensor node waits for the reception of a packet from the other endpoint, it stays with the radio in receive mode, thus consuming a current of *I_rx_*. Once the timer expires, the sensor node turns the radio off and goes through a final postprocessing stage.

Therefore, we can calculate *T_conn_* as follows:

(19) $$T_{conn}=T_{contact}+cSTO+T_{off}+T_{post}$$

where *cSTO* denotes *connSupervisionTimeout* and *I_conn_* can be determined by using the next equation:

(20) $$I_{conn}=\;\frac{I_{adv\_interr}T_{adv\_interr}\;+I_{setup}T_{setup}+N_{CE}I_{avg\_CI}connInterval+I_{rx}cSTO+I_{off}T_{off}+I_{post}T_{post}+2I_{IFS}T_{IFS}}{T_{conn}}$$

where *T_adv_interr_* and *I_adv_interr_* refer to the average duration of the interrupted, last advertising event before connection establishment, and the average current consumed during that time interval. We assume that the initiator may hear any of the *N* advertisements in that last advertising event with the same probability. Therefore, *T_adv_interr_* and *I_adv_interr_* can be computed as shown in the following equations:

(21) $$T_{adv\_interr}=T_{wu}+T_{pre}+\left(\frac{N+1}{2}\right)\left(T_{tx}+T_{tx\_rx}\right)+\left(\frac{N-1}{2}\right)\left(T_{rx}+T_{rx\_tx}\right)$$

(22) $$I_{adv\_interr}=I_{wu}T_{wu}+I_{pre}T_{pre}+\left(\frac{N+1}{2}\right)\left(I_{tx}T_{tx}+I_{tx\_rx}T_{tx\_rx}\right)+\left(\frac{N-1}{2}\right)\left(I_{rx}T_{rx}+I_{rx\_tx}T_{rx\_tx}\right)$$

By plugging Equations (9) and (16)–(22) into Equation (7), the average current consumption of the sensor node in the connection-based approach can be obtained. The sensor node lifetime can be calculated by using Equation (5), but replacing *I_avg_adv_* by *I_avg_conn_*.

#### 4.2.2. Maximum Amount of Collected Data per Contact Interval

We next model the maximum amount of data, denoted *L_data_conn_*, that the mobile entity may collect from the sensor node within a contact interval in the connection-based approach.

Let *L_data_payload_* be the maximum payload size of a data channel packet. Taking into account that only an integer number of round trip exchanges may be performed in each period of *connInterval* size, and considering also that a number of round trip exchanges may be carried out as well in the last connection event (which may have a shorter duration than *connInterval*), *L_data_conn_* can be computed as follows in absence of bit errors:

(23) $$L_{data\_conn}=\left(\left\lfloor \frac{connInterval}{T_{RT}}\rfloor \cdot \lfloor N_{CE}\rfloor +\;\lfloor \frac{\left(N_{CE}-\lfloor N_{CE}\rfloor \right)\cdot connInterval}{T_{RT}}\right\rfloor \right)\cdot \;L_{data\_payload}$$

In a more general scenario where bit errors can take place, and assuming that bit errors are uncorrelated, the previous expression can be rewritten as follows:

(24) $$L_{data\_conn}=\left(\varphi \cdot \lfloor N_{CE}\rfloor +\;\lfloor \varphi \cdot \left(N_{CE}-\lfloor N_{CE}\rfloor \right)\rfloor \right)\cdot \;L_{data\_payload}$$

## 5. Evaluation

In this section, we provide a detailed evaluation of the two introduced approaches for OSDC with BLE, considering the influence of the main BLE parameters and the contact time, for a time between two consecutive contacts of one day. We first use the current consumption measurements on real BLE devices described in the previous section to feed the sensor node current consumption analytical model, for both the advertisement- and connection-based approaches. Results are used to derive the sensor node lifetime in each case. We then compute the theoretical maximum amount of collected data per contact interval, and determine data collection efficiency in terms of energy consumed per collected data bit. We also evaluate and discuss the influence of BER on all considered performance parameters. Finally, we investigate the limitations that real hardware poses to the maximum amount of collected data per interval, by performing experiments. Packet and payload sizes considered for the evaluation are shown in Table 3.

### 5.1. Sensor Node Current Consumption 

#### 5.1.1. Advertisement-Based Approach

**Figure 9:**
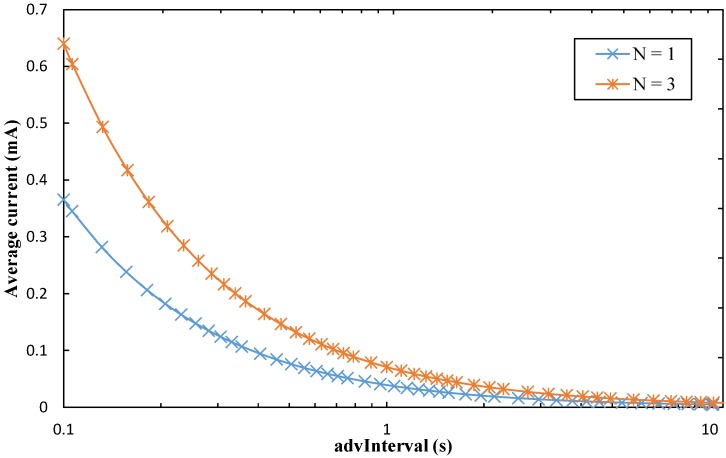
Average current consumption of the sensor node in the advertisement-based approach, as a function of *advInterval*, and for both *N* = 1 and *N* = 3.

By using the values shown in Table 1 and Equations (1)–(4), we calculate the average current consumption of the sensor node in the advertisement-based approach. Figure 9 illustrates the obtained results, as a function of *advInterval*, covering the whole range of values for this BLE parameter. We plot the results for *N* = 1, and for *N* = 3. 

As expected, the average current consumption decreases with *advInterval*, since the sleep intervals have a greater duration for a greater *advInterval*, while the duration of the advertising event remains constant. For the same reason, while for low *advInterval* values the difference between *N* = 3 and *N* = 1 is significant, such difference tends to decrease as *advInterval* increases. 

The average current consumption falls below 26 μA (i.e., the lifetime achievable with a 230 mAh coin cell battery is greater than 1 year, see Section 5.2) for an *advInterval* equal to or greater than 3.06 s and 1.52 s, for *N* = 3 and *N* = 1, respectively. The value of *N* controls a trade-off between sensor node average current consumption and reliability. While using the three advertising channels (*N* = 3) increases current consumption, it may be relevant to face interference or multipath issues, which may occur in urban environments.

Finally, note that sensor node current consumption is independent of the channel BER, as the sensor node will transmit the advertisements in any case (regardless of whether they are correctly received by the mobile entity or not). 

#### 5.1.2. Connection-Based Approach

For the analysis of the connection based-approach, we first study the sensor node average current consumption within a period of *connInterval* (once a connection has been established), we then evaluate the average current consumption during *T_conn_*, and finally we obtain the average current consumption of the connection-based approach.

**Figure 10:**
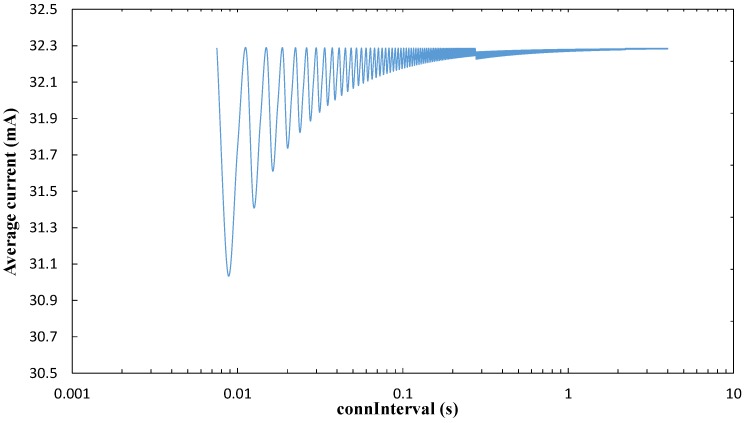
Average current consumption of the sensor node within *connInterval* (*I_avg_CI_*) in the connection-based approach, as a function of *connInterval*, and for BER = 0.

Figure 10 depicts the average current consumption of a sensor during a *connInterval* period (*I_avg_CI_*), as a function of *connInterval*, by using the values in Table 1 and Table 2, and Equation (16). The sawtooth wave of the curve in Figure 10 is due to the fact that only an integer number of round trip exchanges between the two BLE link endpoints may fit in a period of *connInterval* duration. For the lowest *connInterval* value (i.e., 7.5 ms), the number of round trip exchanges that may be performed is 10. As *connInterval* grows, the idle time without round trip exchanges until the end of *connInterval* tends to decrease in relation to the active part of the interval where round trip exchanges take place.

**Figure 11:**
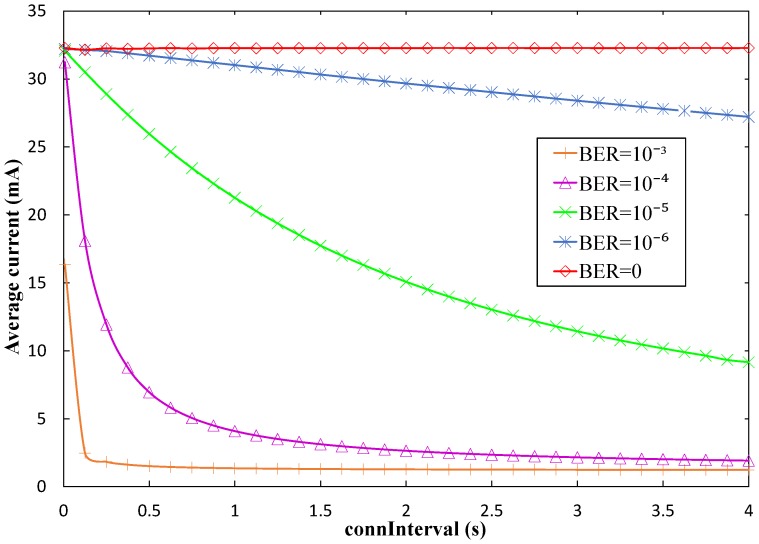
Average current consumption of the sensor node within *connInterval* (Iavg_CI) in the connection-based approach, as a function of *connInterval*, and for several BER values.

Figure 11 illustrates the influence of BER on the same performance parameter, i.e., the average current consumption of a sensor during a *connInterval* period (*I_avg_CI_*). As shown in that figure, current consumption within *connInterval* decreases with the *connInterval* setting (for non-zero BER values), and also decreases with BER. In fact, BLE was not designed for high throughput interactions. When bit errors arise in a connection event, BLE tends to close the connection event quickly and try a different frequency channel for the next connection event (see Section 3.1.2). Once a connection event is closed, the sensor node can sleep until the next one. Sleep interval duration increases with BER and with *connInterval*. This behavior actually saves sensor node energy, but it also reduces the amount of information that can be collected by the mobile entity.

**Figure 12:**
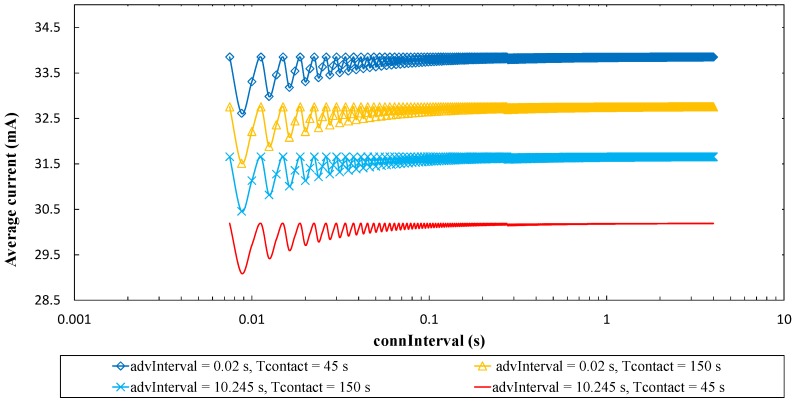
Average current consumption of the sensor node within a *T_conn_* period in the connection-based approach, as a function of *connInterval*, and for *N* = 3 and BER = 0.

We next study the average current consumption during *T_conn_*, for different *T_contact_* values (Figure 12). We assume a *connSupervisionTimeout* of *connInterval*, i.e., the minimum value for this parameter, in order to evaluate the scenario of lowest sensor node energy consumption after connection failure. 

While the sawtooth effect observed in Figure 10 is visible, in Figure 12, such effect is reduced, mainly due to the connection establishment, detection of inactive connection, and connection finalization procedures, which also consume energy.

The *advInterval* parameter may influence the current consumption within *T_conn_*, since the time between the contact start and the next advertising event, during which the sensor node is in sleep mode, may be relevant. For low *advInterval* values, different contact times do not yield noticeable differences in the average current consumption. However, for high *advInterval* values, the difference in the current consumed within *T_conn_* is significant, since *advInterval* becomes significant in comparison with some of the contact times considered (e.g., 45 s). Using different values for *N* leads to negligible differences in the current consumed during *T_conn_*, which are not shown in Figure 12 for the sake of clarity.

**Figure 13:**
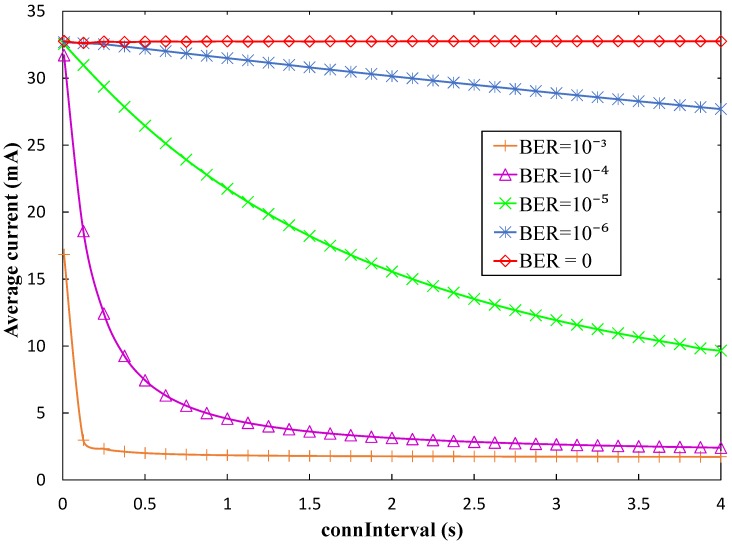
Average current consumption of the sensor node within a *T_conn_* period in the connection-based approach, as a function of *connInterval*, for several BER values, and for *N* = 3, *advInterval* = 0.02 s, and *T_contact_* = 150 s.

We also evaluate the influence of BER on the current consumed by the sensor node over a *T_conn_* period (Figure 13). As an example, we consider *advInterval* = 0.02 s and *T_contact_* = 150 s. A tendency similar to the one observed in Figure 11 is visible, which affects the *connInterval* periods within *T_conn_*. 

**Figure 14:**
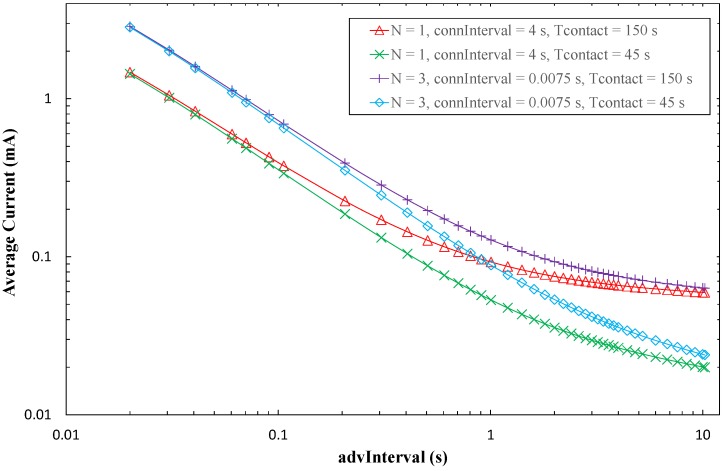
Average current consumption of the sensor node in the connection-based approach, for a time between contacts of one day, as a function of *advInterval*, and for different *N* and *T_contact_*, and for BER = 0. A theoretical value of *T_contact_* = 0 has been evaluated, however depicted results in the logarithmic representation used in the figure are very close to those of *T_contact_* = 45 s. Thus they have been excluded from the figure for the sake of clarity.

Finally, Figure 14 shows the average current consumption of the sensor node, assuming one contact between the sensor node and the mobile entity per day. Average current consumption of the sensor node decreases with *advInterval*, since time between advertising events, during which the sensor node sleeps, increases. For low *advInterval* values, the number of advertising packets sent per advertising event (*N*) determines current consumption, as advertising packet transmission, and subsequent receive intervals for possible incoming Connection Request packets, occur frequently. However, as *advInterval* increases, the relative contribution of the advertising event to the average current consumption decreases. For high *advInterval* values, *T_contact_* is the most relevant parameter, as data exchange during *T_contact_* dominates current consumption over infrequent advertising and sleep intervals between advertising events. Current consumption increases with *T_contact_*, as expected. 

**Figure 15:**
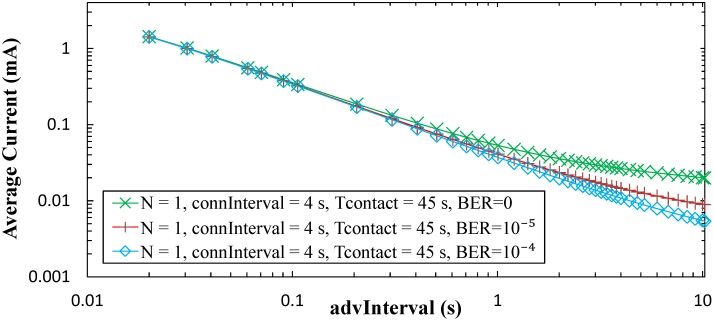
Average current consumption of the sensor node in the connection-based approach, for a time between contacts of one day, for *N* = 1, Tcontact = 45 s, and connInterval = 4 s, as a function of *advInterval*, and for different BER values.

For BER = 0, the *connInterval* setting has a negligible effect on the average current consumption of the sensor for one contact per day. However, for non-zero BER values, *connInterval* becomes relevant. When *connInterval* is low, non-zero BER leads to negligible difference in terms of sensor node average current consumption, compared to a BER = 0 scenario (not shown in Figure 14 for clarity). However, such difference increases with *connInterval*, *advInterval* and BER, and decreases with *N*. For example, Figure 15 illustrates sensor node average current consumption for BER = 10^−4^, BER = 10^−5^, and for the highest *connInterval* value (i.e., 4 s). The sensor node average current consumption difference becomes significant for high *connInterval*, since sleep intervals after connection event termination are then large, and for high *advInterval* and low *N*. The latter happens because the performance difference during *T_conn_* (i.e., the active portion of the time between contacts), as a function of BER becomes more significant when low energy is consumed in the rest of time (in which only advertising activities consume more energy than sleep periods).

#### 5.1.3. Current Consumption Comparison of the Advertisement-Based and the Connection-Based Approaches

**Figure 16:**
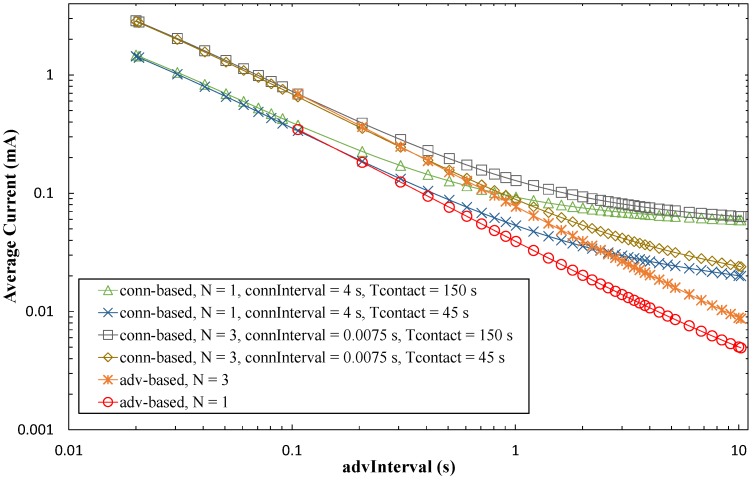
Average current consumption of the sensor node, for the advertisement-based and connection-based approaches, as a function of *advInterval* and for different *N* and *T_contact_* values, and for BER = 0.

In order to ease a comparison between the advertisement- and connection-based approaches, Figure 16 plots the average current consumption of each method, as a function of *advInterval*, and for different *T_contact_* values. For the sake of clarity, only BER = 0 has been considered. Note that, for advertisement-based approaches, the minimum *advInterval* value is 100 ms. 

For a given value of *N*, the corresponding advertisement-based approach has a lower current consumption than connection-based approaches. This happens due to the current consumption of data exchange in the latter, and becomes more significant as *advInterval* increases. Note that current consumption of advertisement-based approaches is independent of *T_contact_*. For the highest *advInterval* values, current consumption of advertisement-based approaches is roughly one order of magnitude lower than that of connection-based approaches.

### 5.2. Sensor Node Lifetime 

Using the average current consumption results obtained in the previous section, it is possible to determine the theoretical lifetime of a battery-operated sensor node. Assuming a button cell battery of 230 mAh (such as the CR2032 model), and the same conditions assumed in the previous section, the sensor node lifetime is plotted in Figure 12, as a function of *advInterval*, and for different contact time values. 

**Figure 17:**
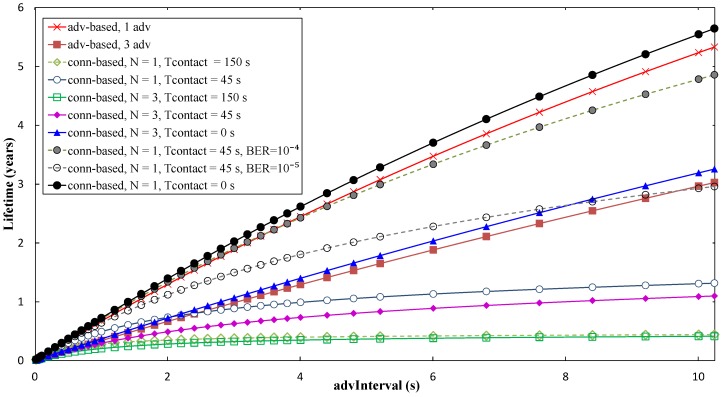
Average sensor node lifetime, for the advertisement-based and connection-based approaches, as a function of *advInterval*, and for different *N*, *T_contact_* and BER values, and assuming a time between contacts of one day. For connection-based results, *connInterval* = 4 s has been assumed.

As shown in Figure 17, with the advertising-based approach, it is possible to achieve a theoretical sensor node lifetime up to 5.33 and 3.02 years, for *N* = 1 and *N* = 3, respectively. Using the connection-based approach, and assuming error-free communication, the sensor node lifetime can be of up to 1.31 and 1.09 years, for *N* = 1 and *N* = 3, respectively, and for *T_contact_* = 45 s. 

A theoretical value of *T_contact_* = 0 s has also been evaluated for the connection-based approach, which provides an upper bound of the achievable sensor node lifetime in this approach. For this setting, the short size of the advertising packet used leads to a current consumption even lower than that of the advertisement-based approaches (which use a greater sized advertising packet) for the same value for *N*. However, sensor node lifetime increases with BER in the connection-based approach, accordingly to the behavior assessed in the previous section. For high *connInterval*, high *advInterval*, and *N* = 1, sensor node lifetime increases by a factor greater than 3 for BER = 10^−4^ compared with a BER = 0 scenario. However, sensor node lifetime increase with BER comes at the cost of lower amount of sensor data collected (see Section 5.3). Sensor node lifetime increases with *advInterval*. However, there exists a trade-off between sensor node lifetime and the amount of sensor data that can be collected during the contact time that depends on *advInterval* (see Section 5.3). For BER = 0, the *connInterval* parameter exhibits a negligible impact on both the average current consumption and the sensor node lifetime. In the presence of channel errors, *connInterval* leads to significant performance difference only for high values for this parameter.

### 5.3. Maximum Amount of Collected Data per Contact Interval 

**Figure 18:**
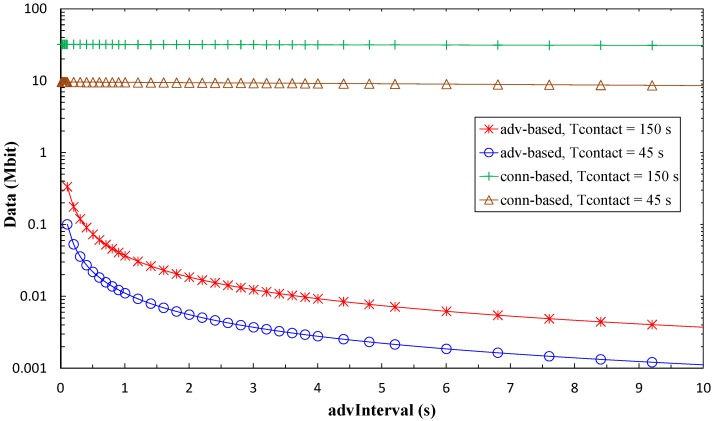
Maximum amount of collected data per contact interval, for the advertisement-based and connection-based approaches, as a function of *advInterval*, and for different *T_contact_* values. Only curves for *connInterval* = 4 s are shown, for the sake of figure clarity.

**Figure 19:**
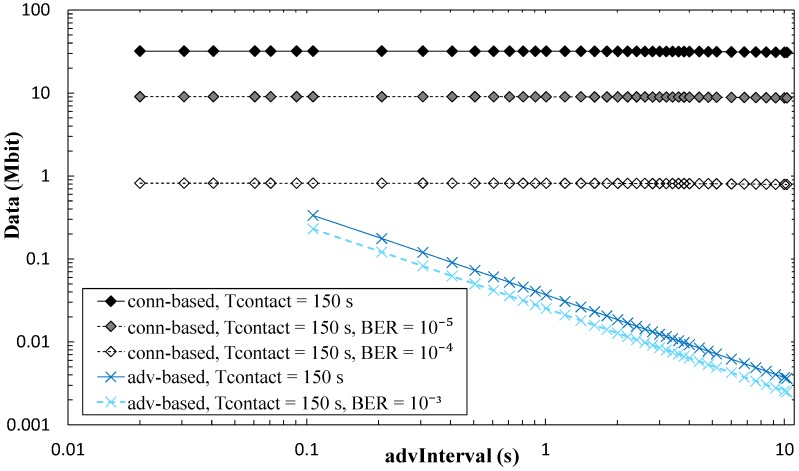
Maximum amount of collected data per contact interval, for the advertisement-based and connection-based approaches, as a function of *advInterval*, for different BER values, and for *T_contact_* = 150 s. *connInterval* = 4 s has been assumed.

By applying Equations (6) and (23), we next compute the theoretical maximum amount of sensor data that can be collected within a contact interval, for both advertisement- and connection-based approaches. Results are shown in Figure 18, as a function of *advInterval* and *T_contact_*, and assuming error-free communication, while impact of non-zero BER is evaluated in Figure 19. Note that the amount of collected data is independent of *N* in the advertisement-based mode (because the same advertising packet is sent through the *N* advertising channels used), and is negligibly affected by *N* in the connection-based mode.

As shown in Figure 18, for low *advInterval* values, the amount of data that can be collected from the sensor node in the advertisement-based approach is 2–3 orders of magnitude lower than that of the connection-based approach. For high *advInterval* values, the difference increases to 4–5 orders of magnitude. In the connection-based mode, the amount of sensor node data that can be collected is almost independent of *advInterval*. With the considered assumptions, if the amount of data to be collected during the contact is below 334 kbit (i.e., ~42 kB), the advertisement-based approach suffices. However, for amounts of data greater than this value, the connection-based approach is the only feasible option, offering a capacity of 10 to 100 Mbit, depending on the contact duration. Considering Figure 17 and Figure 18, a trade-off can be observed between sensor node lifetime and amount of collected data that depends on the *advInterval* setting. 

For the connection-based approach, influence of *advInterval* on the amount of collected data is low, and it decreases as *T_contact_* increases because a greater part of the contact time can be used for data exchange. Increasing *connInterval* slightly improves the amount of collected data. However, in Figure 18, only curves for *connInterval* = 4 s are shown, for the sake of figure clarity. Figure 19 presents results on the impact of non-zero BER on the amount of data collected. As expected, this performance parameter decreases with BER. However, such decrease with BER is greater for the connection-based approach than in the advertisement-based one. In the former, when errors lead to connection event termination, relatively long periods of inactivity (between connection event termination and start of the next connection event) will reduce significantly the amount of collected data. This effect is emphasized for high *connInterval*, where the duration of inactive periods will also be high. In the advertisement-based approach, amount of collected data is only significantly affected by channel errors for BER greater than 10^−4^. We have evaluated BER = 10^−3^, BER = 10^−4^, and BER = 10^−5^, in addition to BER = 0, but only the first one leads to visible differences when compared with BER = 0 in Figure 19.

**Figure 20:**
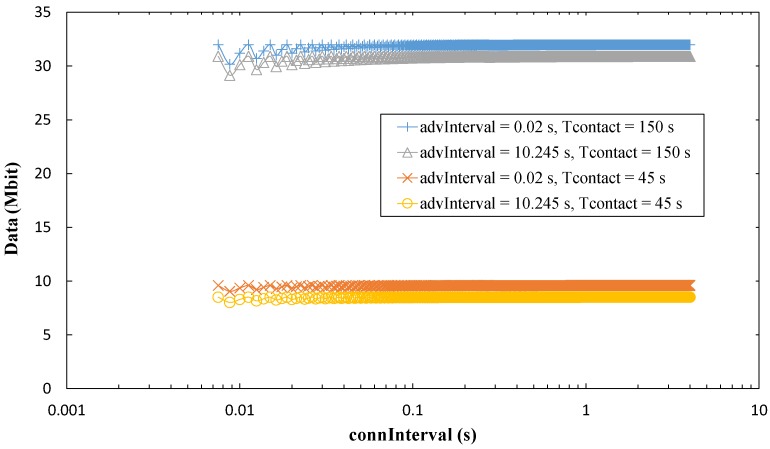
Influence of *connInterval* on the maximum amount of collected data per contact interval, for the connection-based approach, and for different *T_contact_* and *advInterval* values.

**Figure 21:**
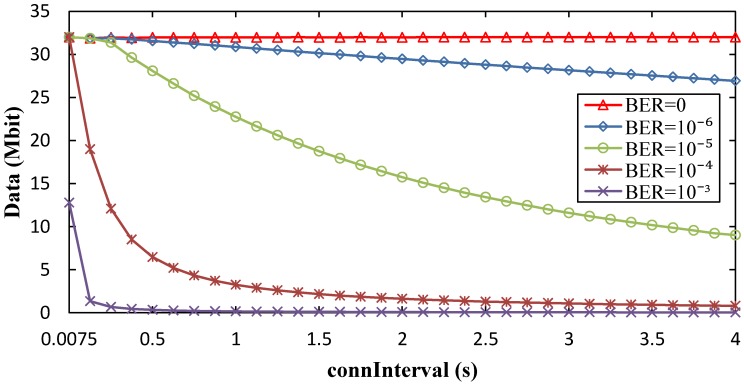
Influence of *connInterval* on the maximum amount of collected data per contact interval, for the connection-based approach, for different BER values, for *advInterval* = 0.02 s and *T_contact_* = 150 s.

We next provide insight on the influence of *connInterval* on the theoretical maximum amount of data collected in the connection-based approach, for BER = 0 and for non-zero values of BER (see Figure 20 and Figure 21, respectively). As shown in Figure 20, for a *T_contact_* of 150 s and 45 s, the amount of collected data tends to grow asymptotically with *connInterval*, since a greater number of round trip exchanges are possible, and the unused part of *connInterval* becomes smaller in relative terms. Fluctuations of the amount of collected data, due to the fact that only an integer number of round trips are allowed per *connInterval*, are mostly significant for *connInterval* values below 100 ms. Figure 21 further illustrates the influence of BER on the amount of collected data as a function of *connInterval*. Amount of collected data decreases with *connInterval* and BER, most significantly for BER greater than 10^−6^. 

### 5.4. Energy Cost 

In the previous two subsections, we have observed that the advertisement-based approach allows to achieve a greater sensor node lifetime (i.e., a lower sensor node energy consumption) than the connection-based one, while the latter provides a greater data transfer capacity. However, for a meaningful analysis, these two performance parameters should be related to each other, to determine the energy cost (in terms of Joules per collected bit) for the two approaches. 

**Figure 22:**
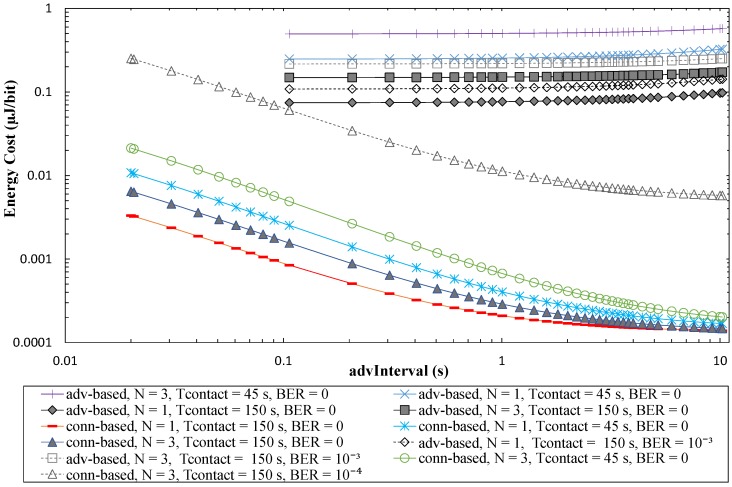
Energy cost for the advertisement-based and the connection-based approaches as a function of *advInterval*, for different *T_contact_* and *N* values, assuming connInterval = 4 s, and a time between contacts of one day.

Figure 22 provides the energy cost for the two approaches, as a function of *advInterval*, and for *T_contact_* values of 150 s and 45 s. In addition, non-zero BER values have been evaluated in some cases to illustrate influence of BER on the energy cost. The energy consumed is determined by first calculating the power consumed (i.e., the average current consumption, computed in Section 5.1, multiplied by the battery voltage assumed, 3 V), and dividing it by the period between two consecutive contacts of one day.

As shown in Figure 22, for low *advInterval* values, the energy cost of the connection-based approach is 1–3 orders of magnitude below that of the advertisement-based approach. The energy cost of the latter tends to increase with *advInterval*, since sleep periods increase and during these no data is collected, but energy is still consumed. However, the energy cost of the connection-based approach decreases significantly with *advInterval*, since there is a large amount of data collected during the contact, while the dominant component in the energy consumed is still the rest of the day, during which the sensor node is advertising its connectability, and the device tends to be mostly in sleep mode (as shown by the average current consumption trend with *advInterval* in Figure 14). The duration of sleep periods increases with *advInterval*.

Figure 22 also shows how non-zero values of BER affect the energy cost for both the advertisement-based and the connection-based approaches. In the former, channel errors reduce the amount of collected data, but do not alter the energy spent in advertisement transmission. Therefore, the energy cost increases with BER, although as found in the amount of collected data analysis, energy cost increase is only significant for BER greater than 10^−4^. In the connection-based approach, channel errors reduce the energy spent by the sensor node, but reduce to a much greater extent the amount of collected data during a connection. In fact, the sensor node consumes a significant amount of energy in periods between contacts (to perform advertisement tasks), while data can only be collected during contact intervals.

### 5.5. Maximum Amount of Collected Data: Experimental Results

Section 5.3 evaluated the maximum amount of collected data per contact interval, following a theoretical method. However, empirical evidence has shown that several factors limit the maximum amount of data that can be transferred via a BLE link in a given time interval [8]. For the sake of a realistic analysis, in this section we conduct experiments in order to determine the achievable amount of collected data per contact interval with the Bluegiga BLE121LR platform. We study the influence of the *connInterval* parameter, as well as the impact of distance between the two BLE devices, on the achievable amount of collected data per contact interval. For the experiments, *connSlaveLatency* has been set to 0, in order to maximize throughput during data collection, while for *connSupervisionTimeout*, the default setting of the platform (i.e., 160 ms) has been used. We consider *T_contact_* values of 45 s and 150 s.

#### 5.5.1. Influence of *connInterval* on Amount of Collected Data per Contact Interval

In order to obtain the amount of collected data per contact interval for different *connInterval* values, two modules are used, as master and slave, respectively. Our goal is to measure the maximum achievable value for the aforementioned performance parameter, at the upper layer Attribute Protocol (ATT) level, i.e., ATT payload per time unit (the reader may refer to the literature for a detailed description of ATT [7,8]). To this end, the slave sends ATT notifications to the master, which does not trigger ATT-layer acknowledgments, at the highest possible rate. The master is connected through a serial link with a PC to trace the BLE Link Layer state of the master, and the amount of successfully transferred packets. For the measurements, a Texas Instruments CC2540 USB sniffer connected to the PC is used to obtain the timestamp of the first and the last packets transferred. The *connInterval* parameter is set in the master. Recall that this parameter is communicated by the master to the slave in the Connection Request message during connection establishment.

To perform the measurements, two different scripts were developed (as a side contribution of this paper, the scripts are both available at [35]). One script generates the data packets sent by the slave, whereas the other is executed at the master for service discovery, connection establishment and data reception. During the tests, the two BLE modules are located at a near-zero distance, in front of each other. A large number of different *connInterval* values are tested within a set of low *connInterval* values, as explained next. For each *connInterval* value, 10 individual experiments are performed. In each experiment, 100 kB of data are transferred.

**Figure 23:**
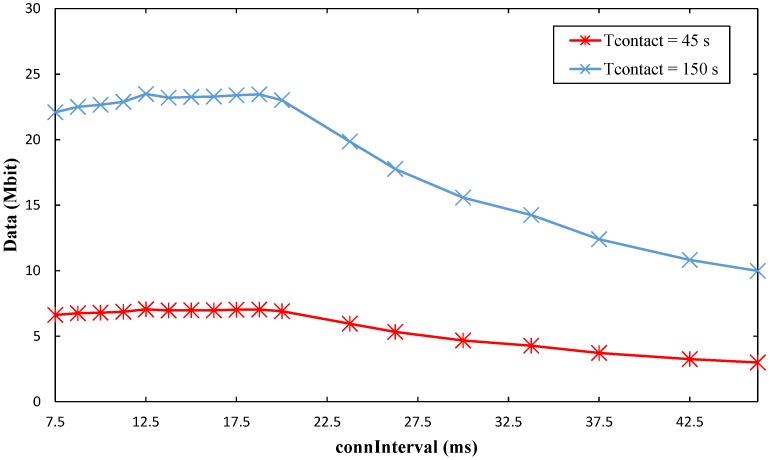
Maximum measured amount of collected data per contact interval, as a function of *connInterval*, for *T_contact_* values of 45 s and 150 s.

As shown in Figure 23, with the lowest *connInterval* values, the amount of collected data increases slightly with *connInterval*, since the idle time in a *connInterval* tends to decrease. As *connInterval* grows, the amount of collected data stays relatively constant up to around *connInterval* = 20 ms. Thereafter, the amount of collected data starts to decrease. 

**Figure 24:**
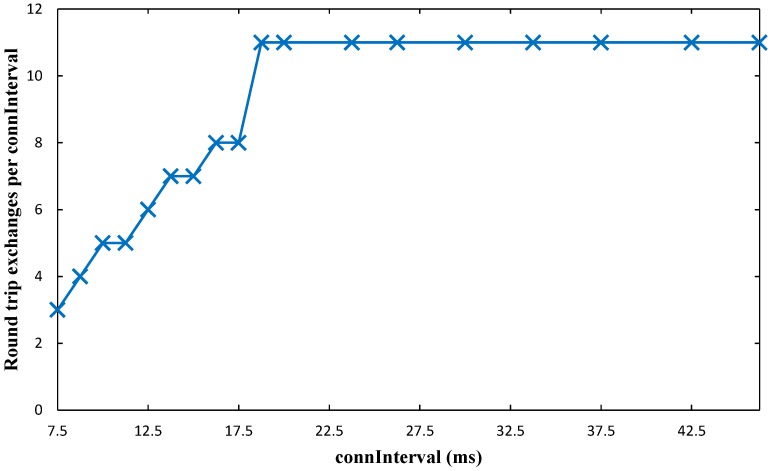
Number of round trip exchanges measured per *connInterval*, as a function of *connInterval*.

In order to provide insight on the above results, we also measured throughput within a contact interval. The maximum throughput within a contact interval achieved during the tests is 156.5 kbit/s. (It must be noted that, to our best knowledge, this obtained throughput value within a contact interval is the highest compared with the works available in the literature (see Section 2.2), and is higher than the one reported by the manufacturer. This was possible by using a timer-based application for generating and sending data, while the example code provided by the manufacturer involves more complex processing, including use of the Analog to Digital Converter (ADC), for the same purpose.) However, this value is limited by the maximum number of round trip exchanges per *connInterval* allowed by the platform, which we found to be 11 (see Figure 24). 

The maximum measured throughput is lower than the maximum theoretical throughput of BLE (reported to be 236.7 kbit/s [30]). This is expected, as BLE implementations are not optimized for high throughput [8,14]. Nevertheless, this fact must be taken into account in a practical OSDC scenario with BLE, where the maximum amount of data that may be collected per contact interval from the sensor node will be lower than the theoretical one. For example, the measured maximum amount of collected data in a contact time of 150 s is 23.5 Mbit, while the theoretical one is around 32 Mbit. 

Finally, because the number of 11 round trips per *connInterval* cannot be exceeded due to radio buffer limitations, increasing *connInterval* beyond 20 ms decreases the amount of collected data per contact interval.

#### 5.5.2. Influence of Distance between the two BLE Devices on the Amount of Collected Data

We next perform experiments to evaluate the effect of the distance between the two BLE modules on the amount of collected data. As aforementioned, to explore the practical limits of OSDC with BLE, we selected the BLE121LR modules for the experiments because they support long range communication, i.e., an extended range in comparison with that of typical BLE platforms. According to the manufacturer data, the maximum range is 450 m [28]. We evaluate the achieved amount of collected data for different distances between the two BLE devices, in two different scenarios: (a) a university campus scenario (UPC Campus del Baix Llobregat, in Castelldefels, Spain); and (b) a beach scenario in the same city. For each considered distance between the two BLE devices, at least 25 individual experiments were carried out. *connInterval* was set to 12.5 ms, as the maximum throughput results in the near-zero distance between the BLE devices were obtained with this value. 

**Figure 25:**
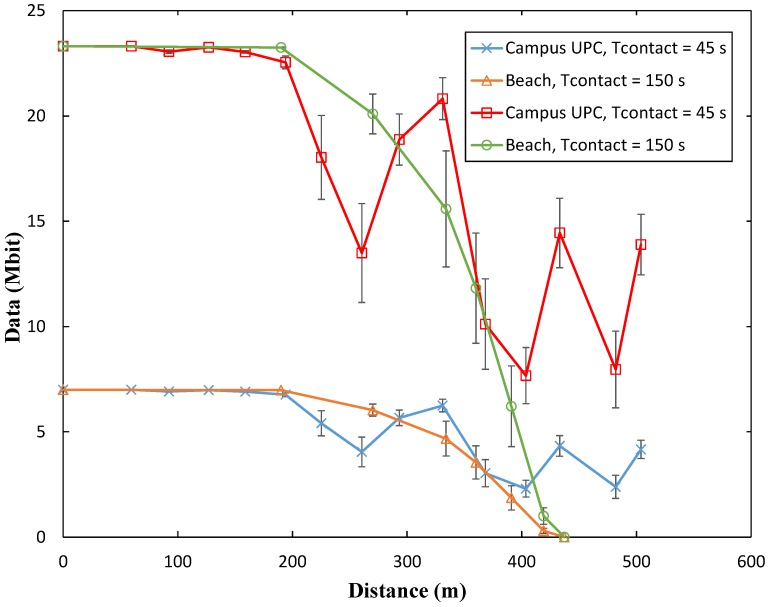
Measured amount of collected data as a function of distance between sender and receiver, in the university campus and beach scenarios.

Figure 25 depicts the average measured amount of collected data, and the corresponding standard deviation, for each considered distance between the two BLE devices. Measurements in the university campus show that in that scenario it is possible to achieve a range beyond the one reported by the manufacturer, probably due to a wave guide effect created by the buildings around the test area. On the other hand, while distance is up to 200 m, the amount of collected data is almost constant, and of similar values as the maximum throughput measured in the previous subsection. Beyond that distance, the amount of collected data initially exhibits a decrease, but from a distance of 300 m it oscillates, which is a characteristic of multipath propagation due to the buildings in the scenario.

Behavior in the beach scenario offers visible differences with the university campus one. While the maximum achievable amount of collected data is the same in both scenarios, in the beach scenario it decreases monotonically for distances between the two BLE devices greater than 200 m, yielding an almost zero amount of collected data already at 437 m.

## 6. Conclusions

BLE has become one of the most adopted technologies in the low-power wireless domain. Given its dominant position in several specific markets, such as smartphones and other consumer electronics devices, BLE is a primary candidate to enable OSDC. 

We have investigated the feasibility and the trade-offs of using BLE as a technology for OSDC from a comprehensive perspective, considering the two main approaches that we have identified (namely, advertisement-based, and connection-based approach), and the main BLE configuration parameters. We have developed analytical current consumption and sensor node lifetime models, derived from the behavior of a real BLE platform, as well as maximum amount of collected data models. We have also studied the energy cost of OSDC, and we have explored hardware limitations on the achievable performance.

Assuming BER = 0, and for a contact time of 45 s, the sensor node lifetime can be as high as 5.3 years and 1.3 years, for the advertisement-based and for the connection-based approaches, respectively, while exploiting the contact interval for data collection. There exists a trade-off with the amount of data that can be collected during the contact interval, which depends on *advInterval*, especially in the advertisement-based approach. If the amount of data to be collected is below ~42 kB, the advertisement-based approach suffices. Otherwise, the connection-based approach should be used, which allows the collection of up to 10 to 100 Mbit. The connection-based approach is between 1 and 5 orders of magnitude more efficient in terms of energy consumed per collected bit than the advertisement-based approach. In the former, influence of *connInterval* on the current consumption and sensor node lifetime is not significant, while an unsuitable setting for this parameter may theoretically decrease the amount of collected data by 13.6%. In the same approach, *advInterval* has a significant impact on the sensor node lifetime, while influence of this parameter on the amount of data that can be collected is limited to up to 22% in relative terms, for the contact time values we have considered.

Channel errors do not affect sensor node lifetime in the advertisement-based approach, therefore favoring sensor node lifetime predictability. In this approach, the energy cost per collected bit increases significantly in the presence of channel errors only for very high BER (e.g., 10^−3^). In the connection-based approach, both current consumption and amount of data collected decrease with BER, although the latter decreases to a greater extent, leading to significant increase of energy cost per collected bit for moderate BER values (e.g., 10^−5^ to 10^−4^), compared with error-free scenarios.

We have also conducted amount of collected data and throughput experiments with a long range BLE platform. We have measured a maximum throughput of 156.5 kbit/s, which can be achieved for *connInterval* values below 20 ms. Radio buffer limitations, among others, do not allow to achieve the maximum theoretical performance of BLE, thus limiting the maximum amount of collected data per connection interval. On the other hand, link delivery performance only decreases for a distance between the two link endpoints beyond 200 m with the platform considered. 

## Figures and Tables

**Table 1:** Definition of states and relevant variables for the advertisement-based approach. In the measurements, the slow clock function offered by the BLE121LR platform was disabled.

State Number	Description	Duration		Current Consumption	
		Variable	Value (ms)	Variable	Value (mA)
1	wake-up	*T_wu_*	0.728	*I_wu_*	9.595
2	radio preparation	*T_pre_*	0.247	*I_pre_*	17.506
3	transmission	*T_tx_*	0.398	*I_tx_*	41.046
4	channel change	*T_ch_*	0.134	*I_ch_*	21.467
5	radio off	*T_off_*	0.190	*I_off_*	10.543
6	postprocessing	*T_post_*	0.818	*I_post_*	10.523
7	sleep	*T_sleep_*	-	*I_sleep_*	1.193 × 10^−3^

**Table 2:** Definition of additional states and relevant variables for the advertiser in the connection-based approach. As the advertisement packet size is different from the one in the advertisement-based approach, details regarding advertisement transmission in the connection-based approach are provided in the table. In the measurements, the slow clock function offered by the BLE121LR platform was disabled.

State Number	Description	Duration		Current Consumption	
		Variable	Value (ms)	Variable	Value (mA)
3	transmission	*T_tx_*	0.229	*I_tx_*	41.046
8	transmit to receive	*T_tx_rx_*	0.106	*I_tx_rx_*	24.952
9	reception	*T_rx_*	0.134	*I_rx_*	29.106

**Table 3:** Packet and payload sizes considered in the evaluation. For a maximum sized data channel PDU, the maximum payload size is 20 bytes of attribute notification. The maximum payload allowed in the non-connectable advertisement type (used in the advertisement-based approach) is 31 bytes.

Packet Type	Packet Size (Bytes)	Payload Size (Bytes)
Data channel PDU (minimum size)	10	0
Data channel PDU (maximum size)	37	20
Advertising channel PDU (non-connectable type, maximum size)	47	31
Advertising channel PDU (connectable type)	23	--
